# The MRI Features and Prognosis of Gliomas Associated With IDH1 Mutation: A Single Center Study in Southwest China

**DOI:** 10.3389/fonc.2020.00852

**Published:** 2020-06-02

**Authors:** Guiquan Shen, Rujia Wang, Bo Gao, Zhongwen Zhang, Guipeng Wu, Whitney Pope

**Affiliations:** ^1^Affiliated Hospital of Guizhou Medical University, Guiyang, China; ^2^Tangshan Gongren Hospital, Tangshan, China; ^3^Binzhou Medical University Hospital, Binzhou, China; ^4^UCLA David Geffen School of Medicine, Los Angeles, CA, United States

**Keywords:** glioma, IDH1 mutation, MRI features, prognosis, overall survival

## Abstract

**Purpose:** To investigate the associations of MRI radiological features and prognosis of glioma with the status of isocitrate dehydrogenase 1 (IDH1).

**Material and Methods:** A total of 116 patients with gliomas were retrospectively recruited from January 2013 to December 2015. All patients were undergone routine MRI (T1WI, T2WI, T2-FLAIR) scanning and contrast-enhanced MRI T1WI before surgery. The following imaging features were included: tumor location, diameter, the pattern of growth, boundary, the degree of enhancement, mass effect, edema, cross the middle line, under the ependyma. χ^2^ and Fisher's exact probability tests were used to determine the significance of associations between MRI features and IDH1 mutation of glioma. The survival distributions were estimated using Kaplan-Meier compared by Log-rank test. Univariate and multivariate analyses were performed using Cox regression.

**Results:** Gliomas with IDH1 mutant were significantly more likely to exhibit homogeneous signal intensity (*p* = 0.009) on non-contrast MRI protocols and less contrast enhancement (*p* = 0.000) on contrast enhanced T1WI. IDH1 mutant type glioma was more inclined to cross the midline to invade contralateral hemisphere (*p* = 0.001). The overall survival between IDH1 mutated and wild type glioma were significantly different (*p* = 0.000), age ≤ 40 (*p* = 0.003), KPS scores > 80 before operation (*p* = 0.000) and low grade glioma (*p* = 0.000).

**Conclusions:** Our results suggest IDH1 mutant in gliomas is more likely to exhibit homogeneous signal intensity, less contrast enhancement and more inclined to cross the midline. Patients with IDH1 mutated, age ≤ 40, KPS scores > 80 before operation and low-grade glioma may have a longer life and better prognosis.

## Introduction

Glioma is the most common intracranial tumor in the central nervous system (CNS). According to the Central Brain Tumor Registry of the United States (CBTRUS), the proportion of gliomas in CNS tumors is about 27%, accounting for 80% in the primary malignant tumors (1). High-grade glioma is more malignant which has a more aggressive growth pattern than low grade glioma. This leads to unsatisfactory results after treatment which consists of radiotherapy, chemotherapy and combination therapy. More recent work suggests that we could differentiate tumors of the same WHO grade and morphologic type by using of molecular data to realize the goal of personalized medicine (2).

The 2016 WHO Classification criteria which combined histology with molecular phenotype, such as IDH1 (isocitrate dehydrogenase-1), 1p/19q, BRAF, ATRX (3). Several molecular biomarkers, including IDH1 (4), MGMT (5), epidermal growth factor receptor [EGFR]), may be associated with overall survival of patients with GBM (6). Grant et al. (7) noted MGMT promoter methylation, 1p/19q codeletion, and IDH1 mutations are useful molecular biomarkers for characterizing status of glioma. IDH1/2 mutation was first reported by Parsons et al. (8). IDH1/2 is a key enzyme in the process of tricarboxylic acid cyclic metabolism. IDH1/2 are mainly found in II, III astrocytoma and oligodendroglioma, but rarely in primary glioblastoma or pilocytic astrocytoma (9). In addition, IDH1 has the mutation of homologous gene, IDH2 mutation was also found in glioma. However, IDH1 mutation is more common than IDH2 mutation (10). In previous studies the survival period of IDH1 mutant was significantly different from that of the IDH1 wild type (11). Different phenotypes of genes may be related to different portions of glioma. IDH1 wild type glioma was mainly found in GBM, but it could be found in the temporal lobe and had a large volume in WHO II glioma. The correlation between MRI features and gene phenotypes in oligodendroglioma, oligodendrocytoma and GBM have been reported (12–15). There was a correlation between MRI features and expression of gene phenotype of glioma. The purpose of this study is to investigate the relationships between IDH1 mutation and MRI features as well as prognosis in patients with glioma.

## Materials and Methods

### Subjects

The institutional ethics committees of our institution approved the study and granted informed consent. From January 2013 to December 2015 a total of 135 cases of glioma were diagnosed by pathology in The Affiliated Hospital of Guizhou Medical University. All patients underwent routine MRI (T1WI, T2WI, T2-FLAIR) and contrast enhanced MRI before neurosurgery. Imaging features of tumor include: location, diameter, pattern of growth, boundary, degree of enhancement, mass effect, edema, cross the middle-line, under the ependyma.

Inclusion criteria: (1) The clinical and imaging data are complete and reliable; (2) No other adjuvant therapy such as radiotherapy or chemotherapy was performed before operation; (3) It is diagnosed glioma by histopathology after surgery; (4) Important organs (heart, liver, kidney, etc.) function basically normal; (5) Complete data were obtained for follow-up. Exclusion criteria: (1) The patient had a history of other malignancies; (2) The patient had related post-operative complications, such as intracranial hematoma, intracranial infection; (3) Patients who died from other diseases. According to the inclusion and exclusion criteria, totally 116 patients were recruited in this study and associated data of all patients was collected. Among them, 55 males and 61 females had been included. The range of age was from 18 to 86 years old, including 56 low-grade and 60 high-grade.

### IDH1 Mutation Detection

PCR (nested methylation-specific PCR) methods were used to detect the status of IDH1 mutation. There were 62 IDH1 mutant and 54 IDH1 wild type in glioma. The status of IDH1 mutation was detected by direct sequencing and PCR. IDH2 mutations were not detected. The sequence of sequencing was compared with the original sequence of IDH1 to analyze whether the specific base position was mutated. According to the operation manual of EZNA Tissue DNA Kit (OMEGA, America), DNA was extracted from the tumor tissue. DNA purity and content were extracted by spectrophotometer. Primers were designed according to IDH1 genome sequence, upstream primer sequence: 5′-CGGTCTTCAGAGAAGCCATT-3, downstream primer sequences: 5′-GCAAAATCACATTATTGCCAAC-3. Annealing temperature is 60°C, the length of PCR product was 129bp.

### Protocols

All brain MRI examinations were performed on Philips 3.0T MRI Scanner, using 8-channel SENSE head coil. Each patient underwent routine MRI and enhanced MRI before neurosurgery, including axial T1-weighted imaging (T1WI), sagittal T2 weighted imaging (T2WI), axial T2-weighted fluid attenuated inversion recovery (T2-FLAIR) and contrast-enhanced T1-weighted imaging (T1WI+C). Scanning parameters: (1) Axial T1WI: TR 2,270 ms, TE 20 ms, FOV 196 mm × 196 mm, matrix: 288 × 190, NEX: 2, slice thickness:6 mm, slice gap:1 mm. (2) Axial and sagittal T2WI: TR 2,500 ms, TE 90 ms, FOV 230 mm × 230 mm, matrix 420 × 306, NEX:2, slice thickness:6 mm, slice gap:1 mm. (3) Axial T2-FLAIR:TR 8,000 ms, TE120 ms, FOV 230 mm × 230 mm, matrix: 304 × 216, NEX: 2, slice thickness: 6 mm, slice gap: 1 mm. (4) T1WI+C: TR 200 ms, TE 2 ms, FOV 230 mm × 230 mm, matrix 256 × 256, NEX: 2, slice thickness: 6 mm, slice gap:1 mm. The enhanced scan was injected with Gadopentetate Dimeglumine (Gd-DTPA) with a dose of 0.1 ml/kg body weight and injection rate of 3 ml/s, the high-pressure injector was not used.

### Clinical Follow-Up

A retrospective analysis of 132 cases was made, but 16 cases were excluded by inclusion and exclusion criteria. The ways of follow-up consist of consulting inpatient medical records, telephone call inquiry and questionnaire. The content of follow-up includes post-operative survival (death or survival): if the patient survived, his/her physical conditions post-operative radiotherapy and chemotherapy was recorded; if the patient died, the exact cause of death was asked for.

### Statistical Analysis

The data analysis was performed using SPSS19.0 package. χ^2^ test and Fisher's exact probability test were used to analyze the correlation between MRI features of glioma and IDH1 mutation. The survival distributions were estimated using Kaplan-Meier and compared by Log-rank test. Univariate and multivariate regression analyses were performed using Cox proportional hazards regression model. *P* < 0.05 was considered to indicate statistically significant.

## Results

### Correlation Between IDH1 Mutation and MRI Features of Glioma (Table 1, Figures 1, 2)

**Figure 1:**
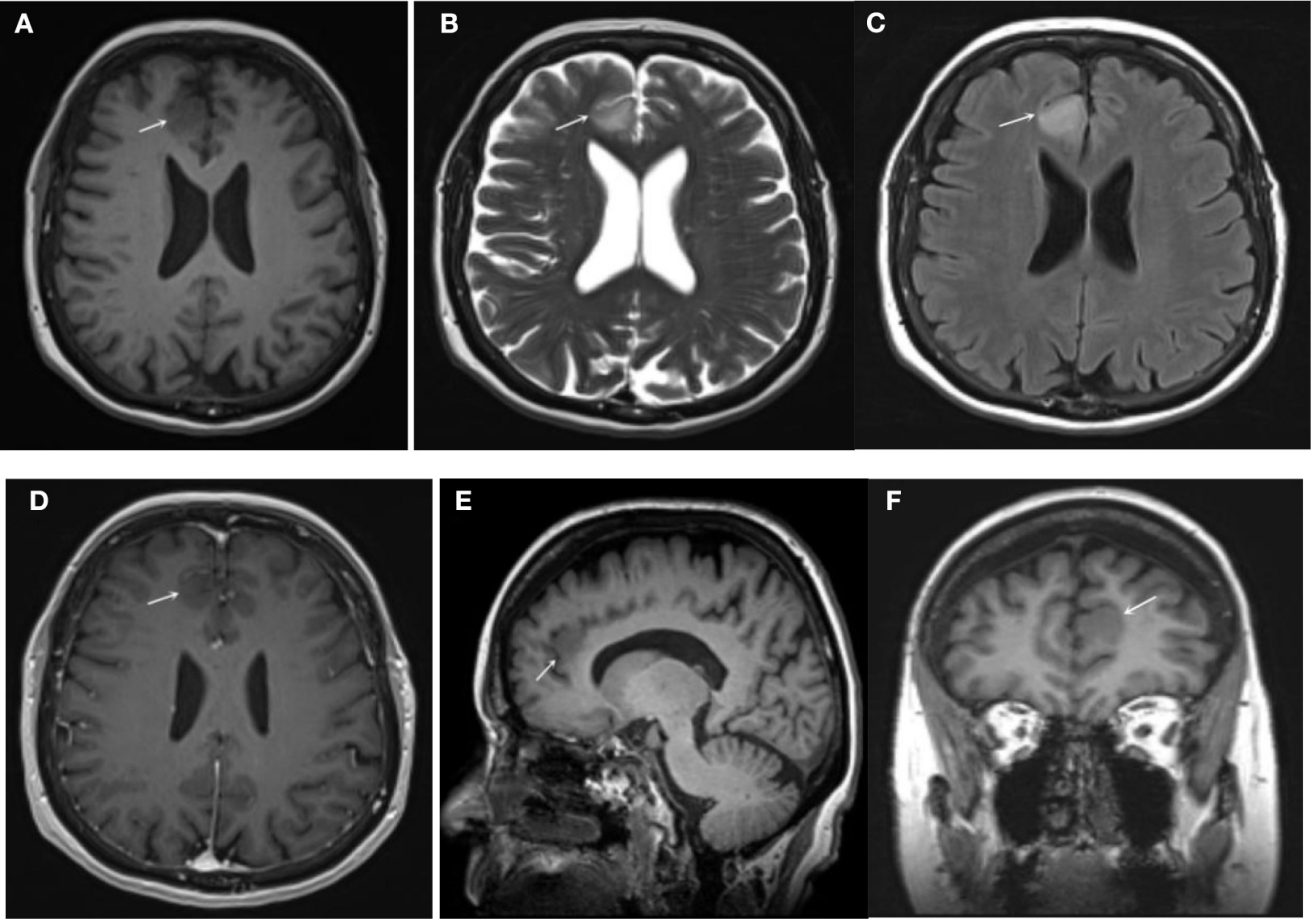
IDH1 mutated glioma (low grade): **(A)** T1WI **(B)** T2WI **(C)** FLAIR **(D)** T1+C (axial) **(E)** T1+C (sagittal) **(F)** T1+C (coronal). The lesion had a clear border, homogeneous signal intensities on T1WI and T2WI, as well as on FLAIR. The lesion presented mild homogeneous enhancement on post-contrast T1WI.

**Figure 2:**
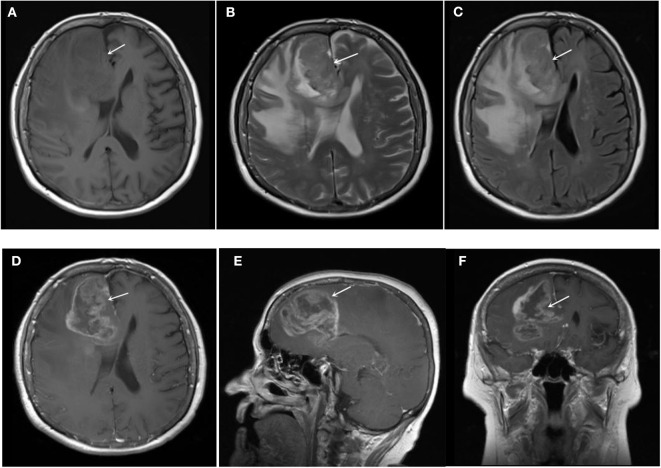
IDH1 wild-type glioma (low grade): **(A)** T1WI **(B)** T2WI **(C)** FLAIR **(D)** T1+C (axial) **(E)** T1+C (sagittal) **(F)** T1+C (coronal). The lesion had unclear border, heterogeneous signal intensities on T1WI, T2WI, and FLAIR. The lesion presented markedly heterogeneous enhancement on post-contrast T1WI.

This study included 116 cases of glioma. Among them, 61 cases of low-grade glioma, 55 cases of high-grade glioma, 62 cases of IDH1 mutant glioma (Figure 1) and 54 cases of IDH1 wild-type glioma (Figure 2). IDH1 mutant glioma was significantly more likely to exhibit homogeneous signal intensity (*p* = 0.009) and less contrast enhancement (*p* = 0.000) on MRI. IDH1 mutant glioma was more likely to cross the midline to the other hemisphere (*p* = 0.001).

**Table 1:** Correlation between IDH1 mutation and MRI features of glioma.

**MRI features**	**All**	***P*-value**	**Low** **grade**	***P*-value**	**High** **grade**	***P*-value**
**Pattern of growth**						
Unilateral	61/113	0.902	51/55	0.929^*^	10/58	0.692^*^
Bilateral	1/3		1/1		0/2	
**Tumor margins**						
Sharp	13/25	0.869	13/14	0.549	0/11	0.2326
Indistinct	49/91		39/42		10/49	
**Signal intensity**						
Homogeneous	46/20	0.009	16/17	0.747	0/3	0.573^*^
Heterogeneous	16/96		36/39		10/57	
**Contrast enhancement**						
Absent or slight	48/57	0.000	46/50	0.627^*^	2/7	0.719
Significant	14/59		6/6		8/53	
**Mass effect**						
Absent or moderate	24/41	0.416	23/26	0.504	1/15	0.424
Severe	38/75		29/30		9/45	
**Edema**						
Absent or moderate	27/47	0.476	26/28	0.604	1/19	0.215
Severe	35/69		26/28		9/41	
**Cross the midline**						
Yes	4/8	0.001	3/3	0.797^*^	1/5	1.000^*^
No	58/108		49/53		9/55	
**Under the ependyma**						
Yes	33/55	0.115	23/25	0.765	10/30	0.001
No	29/61		29/31		0/30	
**Diameter of tumor**						
≥5 cm	24/46	0.978	18/19	0.874	6/27	0.486
<5 cm	38/70		34/37		4/33	

* *Fisher's exact probability test*.

### Relationship Between IDH1 Mutation and Prognosis of Glioma (Table 2, Figure 3)

Survival distributions were estimated using the Kaplan-Meier method. Log-rank test was used for correlation analysis. The overall survival of patients with pre-operative KPS score > 80, age ≤ 40 were significantly longer than patients with KPS <80, age > 40 (Figure 4). The overall survival of low grade glioma was significantly longer than overall survival of high grade glioma.

**Table 2:** Factors affecting the prognosis of glioma.

**Factor**	**Cases (%)**	**Cox univariate analysis**		**Cox multivariate analysis**	
		**HR (95% CI)**	***P^***a***^-*value**	**HR (95% CI)**	***P^***b***^-*value**
**IDH1**					
Wild-type	54 (46.6)	Ref		Ref	
Mutant-type	62 (53.4)	0.205 (0.1, 0.419)	0.000	0.494 (0.126, 1.942)	0.313
**Age**					
>40	37 (31.9)	Ref		Ref	
≤ 40	79 (68.1)	3.67 (1.569, 8.586)	0.003	1.546 (0.24, 9.974)	0.647
**Gender**					
women	61(52.6)	Ref		–	–
men	55 (47.4)	0.812 (0.475, 1.385)	0.444	–	–
**KPS score**					
≥80	41 (35.3)	Ref		Ref	
<80	50 (43.1)	5.554 (2.495, 12.361)	0.000	7.579 (2.802, 20.502)	0.000
**WHO grade**					
Low grade	56 (48.3)	Ref		Ref	
High grade	60 (51.7)	7.98 (3.175, 20.058)	0.000	4.2 (0.481, 36.656)	0.194

**Figure 3:**
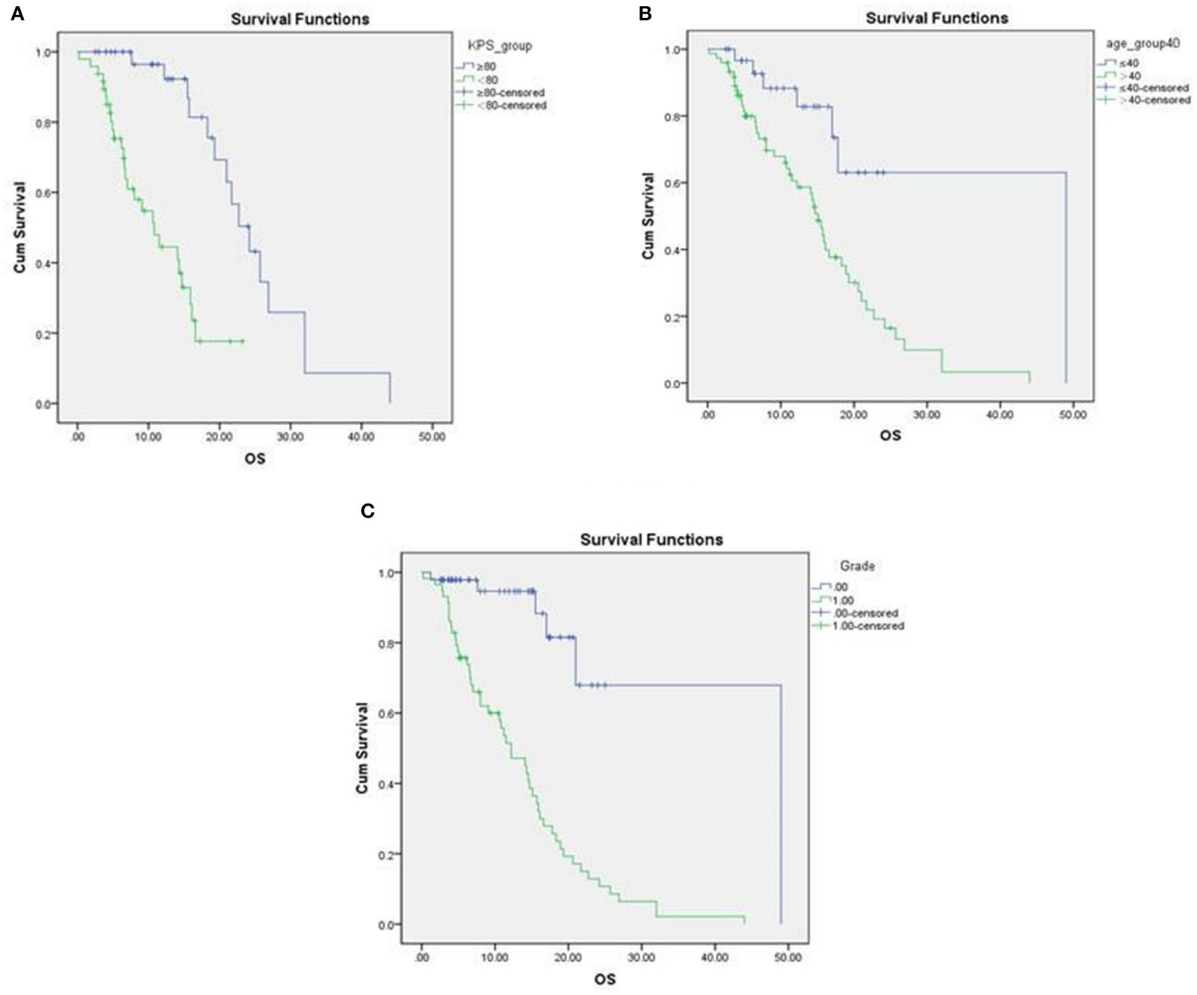
Survival curves associated with preoperative KPS scores, age and grade of glioma. **(A)** The overall survival of the preoperative KPS score <80 was significantly longer than the KPS score <80; **(B)** Comparing the age was <40 with the age was > 40 years old, the difference was statistically significant. The former has a longer life; **(C)** The overall survival of low-grade glioma and overall survival of high-grade glioma were compared. The low-grade glioma had a longer life.

**Figure 4:**
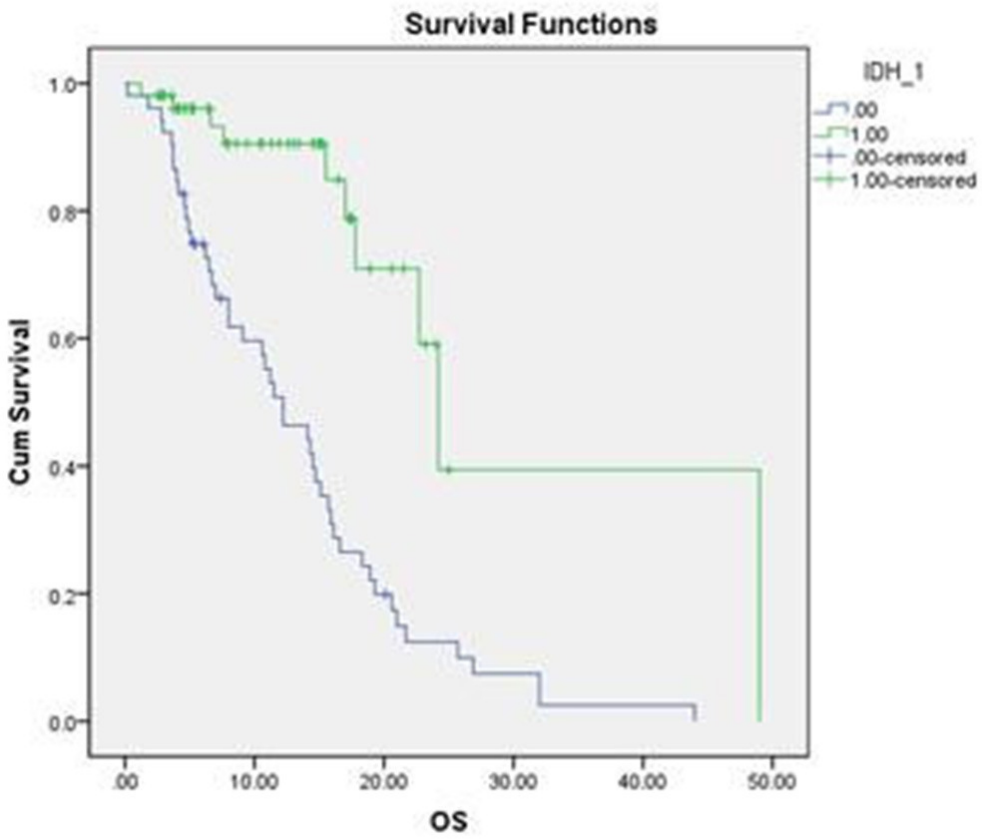
Survival curves associated with IDH1 mutation. The overall survival of IDH1 mutant glioma was significantly longer than IDH1 wild-type glioma.

### Difference Between OS of IDH1 Mutant Glioma and IDH1 Wild Type Glioma (Figure 4)

The difference between the OS of IDH1 mutant glioma and IDH1 wild type glioma were significant, which was statistically significant. Hazard ratio (HR) was 0.205, 95% Confidence interval (CI) was (0.1, 0.049). Compared with the OS of patients >40 and ≤ 40 were significantly different, HR was 3.67, and 95% CI was (0.1, 0.049). The overall survival of the preoperative KPS score > 80 were significantly different from that of KPS score <80. HR was 5.554, 95% CI was (2.495, 12.361). The overall survival of low-grade glioma and overall survival of high-grade glioma were compared, the difference was statistically significant. Cox proportional risk regression multiple factor analysis. The overall survival of the preoperative KPS score > 80 were significantly different from that of KPS score <80. HR was 7.759, 95% CI was (2.802, 20.502). The above results were consistent with the Cox proportional risk regression single factor analysis.

## Discussion

We found IDH1 mutant glioma were inclined to cross the midline to the other hemisphere and were more likely to exhibit homogeneous signal intensity as well as less contrast enhancement. The findings are compatible with IDH1 wild type glioma being more aggressive than IDH1 mutant type. Although these features were not significantly different between low and high grade subgroups, which may be related to the small sample size of this study. A previous study (16) reported IDH1 mutations could reduce the pericyte coverage of microvessels in astrocytic tumors by inhibiting the expression of angiogenesis factors. Feyissa et al. (17) found glioma-related preoperative seizures and post-operative seizure control may be associated with IDH1 mutation but no other characteristic findings such as location, grade or histopathology. Metellus et al. (18) found that a correlation between the location of the tumor and the phenotype in oligodendroglioma, oligocytoma and GBM. Yu et al. (19) found an association between the anatomical location and IDH1 mutation status in low grade gliomas. However, the mechanism of IDH1 mutation and the significance of prognosis in tumor growth were still unclear. The larger sample is warranted to investigate the potentially possible mechanism.

This study found the overall survival period of IDH1 mutated glioma was significantly different from that of IDH1 wild-type glioma. It was reported high grade gliomas with IDH1 mutations had a longer survival period compared to those with IDH1 wild type (20, 21). Van den Bent et al. (22) found there was no relationship between the prognosis of IDH1 mutation glioma and chemotherapy drug administration in a randomized study. In another retrospective study, there was no correlation between prognosis of IDH1 mutant and chemotherapeutic drugs for gliomas. All the above studies have shown the prognosis of IDH1 mutant glioma was better because of its lower grade biological behavior, rather than the treatment effect of chemotherapy itself.

Previous studies have shown age is an independent factor in the prognosis of glioma. Most of the literatures has reported the duration of survival of glioma patients was negatively correlated with age (23). The reason for the prognosis of patients may be that with the increase of age, the metabolism, regeneration, compensatory and immune function of the middle-aged and elderly patients are worse than those of younger patients with glioma. Preoperative KPS score is an evaluation index for patients' overall functional status. Zinn et al. (24) believed that patients with KPS score above 70 or 80 could have a better prognosis (25). The higher the preoperative KPS score, the better the functional status of patients, the better the tolerance to surgery, radiotherapy and chemotherapy. In addition, Goyal and others found qualitative diffusion signature is an adjunct to contrast enhanced MRI, which may has the widest potential impact on clinical care for patients with recurrent high-grade gliomas (26). Bangalore et al. found that high IDH classification accuracy using only T2-weighted MR images by voxelwise deep-learning IDH classification network which showed a high accuracy of 97% in predicting IDH mutant status in gliomas. This represents an important milestone toward clinical translation.

This study has some potential limitations for its single center study. The relatively small sample number may lead to the weakness of statistical significance. It ought to group gliomas into different grades to investigate the relationship between IDH1 mutation and prognosis. This study is a retrospective study; it is difficult to maintain consistency of specific therapeutic regimens. Due to the lack of IDH2 results, the positive of the study may be reduced. In addition, We did not test other markers in this study. we did not have any other multi-model MRI scans. In the furture we will do Multi-model MRI scans to investigate mechanism and prognosis in glioma.

## Conclusions

Gliomas with IDH1 mutations are more likely to exhibit homogeneous signal intensity, less contrast enhancement and are more likely to cross the midline to the other hemisphere. Patients with IDH1 mutated, age ≤ 40, KPS scores > 80 before operation and low-grade glioma may have a longer life and a better prognosis.

## Data Availability Statement

The data analyzed in this study was obtained from the Affiliated Hospital of Guizhou Medical University. Requests to access these datasets should be directed to BG, gygb2004@163.com.

## Ethics Statement

The studies involving human participants were reviewed and approved by the Human Ethics Committee of The Affiliated Hospital of Guizhou Medical University. The patients/participants provided their written informed consent to participate in this study. Written informed consent was obtained from the individual(s) for the publication of any potentially identifiable images or data included in this article.

## Author Contributions

RW, BG, and GS reviewed and drafted the manuscript. RW did the literature search. GW and ZZ modified the figures. WP have revised and edited the manuscript.

## Conflict of Interest

The authors declare that the research was conducted in the absence of any commercial or financial relationships that could be construed as a potential conflict of interest.
